# Increasing Provasculature Complexity in the Arabidopsis Embryo May Increase Total Iron Content in Seeds: A Hypothesis

**DOI:** 10.3389/fpls.2017.00960

**Published:** 2017-06-08

**Authors:** Hannetz Roschzttardtz, Sofía Bustos, Maria F. Coronas, Miguel A. Ibeas, Susana Grant-Grant, Joaquín Vargas-Pérez

**Affiliations:** Departamento de Genética Molecular y Microbiología, Facultad de Ciencias Biológicas, Pontificia Universidad Católica de ChileSantiago, Chile

**Keywords:** iron, embryo, endodermis, provasculature, biofortification

## Abstract

Anemia due to iron deficiency is a worldwide issue, affecting mainly children and women. Seed iron is a major source of this micronutrient for feeding, however, in most crops these levels are too low to meet daily needs. Thus, increasing iron allocation and its storage in seeds can represent an important step to enhance iron provision for humans and animals. Our knowledge on seed iron homeostasis is mainly based on studies performed in the model plant *Arabidopsis thaliana*, where iron accumulates in endodermis cells surrounding the embryo provasculature. It has been reported that cotyledon provasculature pattern complexity can be modified, thus we hypothesize that changes in the complexity of embryo vein patterns may affect total iron content in Arabidopsis seeds. This approach could be used as basis to develop strategies aimed to biofortify seeds.

## Introduction

Malnutrition is one of the greatest health problems in the world, causing chronic diseases, altered physical and mental development, as well as reduced socioeconomic development of the countries most affected. The major problem of micronutrient deficiency worldwide is iron deficiency, which is a serious public health problem and a major concern for the World Health Organization (WHO). According to WHO, 30% of the world’s population suffers anemia, affecting mainly women and children. Also, the role of iron in fertility and seed yield is an important agronomical trait, because low iron bioavailability in soils, widespread in arable soils, limits plant fertility (Guerinot and Yi, 1994). Recent evidence shows that the amount of micronutrients in seeds has declined systematically since the beginning of the so-called green revolution in the 1960s (Fan et al., 2008; DeFries et al., 2015). Crops biofortification is an agronomic tool that can be implemented to solve malnutrition by increasing the natural content of micronutrients in plants. Biofortification can improve the nutritional content of staple foods that are consumed by the population, resulting in a cost-efficient and sustainable way of delivering nutrients to poor or rural populations with limited access to markets or health centers that provide fortified foods or supplements. To modify the iron content of seeds for human consumption (biofortification) is an attractive alternative to combat iron deficiency, however, mineral loading of seeds is strictly controlled in plants and for instance an increase in root absorption does not produce necessarily seeds with higher mineral content (Waters and Grusak, 2008; Murgia et al., 2012). Environmental factors and transport activity may be also limiting seed Fe content. Recently, a mechanism depending on ascorbate efflux in plant embryos has been described and it could play a role in the control of iron loading in seeds (Grillet et al., 2014). However, still little is known about transport activity and iron content in seeds. In addition to metabolic and physiological approaches, plant anatomy need to be tackled in order to reach micronutrient improvement in plants (Vasconcelos et al., 2016).

## Iron Distribution in Arabidopsis Embryo

**FIGURE 1:**
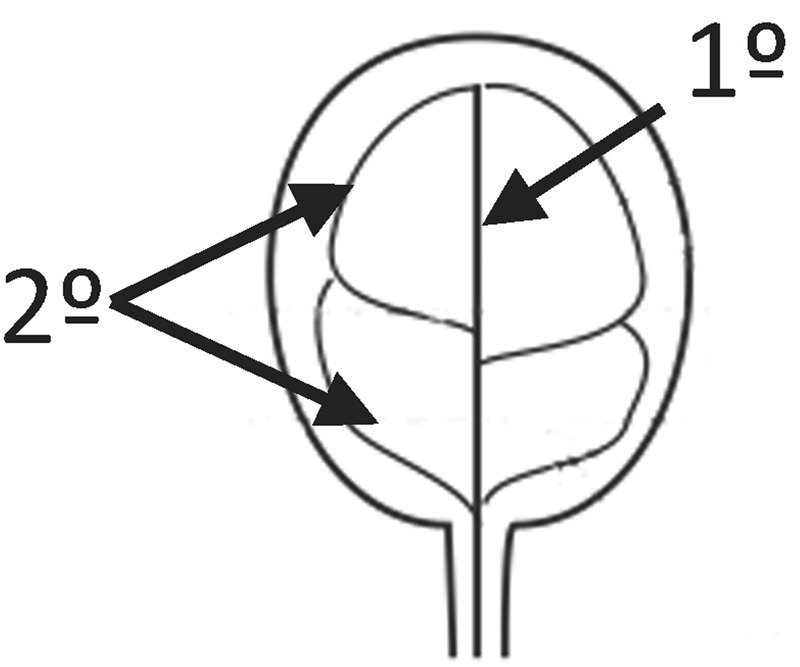
Provasculature patterns in Arabidopsis cotyledons. In the figure 1° indicates the primary vein and 2° secondary veins. 1° and 2° are forming 4 areoles in this case.

**FIGURE 2:**
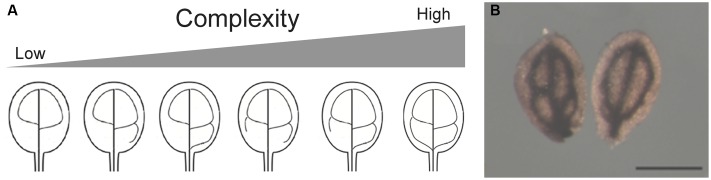
**(A)** Arabidopsis cotyledon provasculature complexity. Different provasculature patterns found in wild type embryos are shown. **(B)** Cotyledons with different provasculature complexities have the same size. Mature wild-type cotyledons were stained with Perls/DAB to detect iron accumulation around the provasculature. Bar, 50 μm.

**FIGURE 3:**
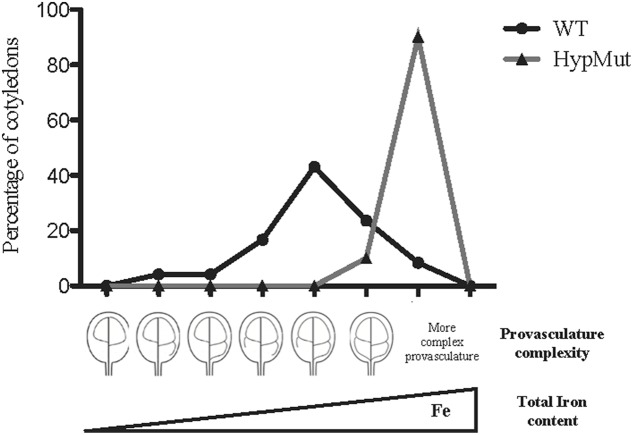
Hypothetic model indicating that Fe total content in seeds will increase in a population of embryos with maximal provasculature complexity. WT data is from Roschzttardtz et al. (2014). HypMut correspond to a hypothetical seed population containing cotyledons with higher provasculature complexity.

Elemental imaging of metals using Arabidopsis seeds were previously obtained by X-ray tomography fluorescence (XRF) and Perls/DAB staining: iron accumulates in the vacuoles of the endodermis/perivascular cell layer during *Arabidopsis thaliana* seed maturation (Kim et al., 2006; Roschzttardtz et al., 2009; Mary et al., 2015). Endodermis cells surround provasculature and iron can be used as a marker of provasculature patterning in Arabidopsis embryos (Roschzttardtz et al., 2010, 2014). By the time seeds reach maturity the embryo has developed a complete array of procambial strands. After germination, the embryonic provasculature strands differentiate into vascular bundles consisting mostly of phloem and xylem (Sieburth and Deyholos, 2006). In Arabidopsis, the vein pattern in embryo cotyledons is simple, consisting in primary and secondary veins that can be connected forming areoles (**Figure 1**). Embryo provasculature cotyledon pattern categories can be defined using Perls/DAB staining, and different degrees of pattern complexity can be found in cotyledons from wild type seeds (**Figures 2A,B**). Simplest complexity pattern can have only two areoles while four and more areoles are found in the cases of higher complexity (**Figures 2A,B**). Interestingly, in a wild type seed population, cotyledons with different provasculature complexity patterns are found (**Figures 2A**, **3**). These categories also can be defined in cotyledons by visualizing lignin autofluorescence from mature xylem cells in 1-week-old seedlings (Cnops et al., 2006; Truernit et al., 2012; Roschzttardtz et al., 2014). Different reports indicate that provasculature vein pattern can be modified either decreasing or increasing its complexity (Carland et al., 2002, 2016; Sieburth et al., 2006; Truernit et al., 2012; Roschzttardtz et al., 2014). Noteworthy, a change in provasculature complexity does not lead to changes in cotyledon size (**Figure 2B**). Considering that cotyledon veins are surrounded by the endodermis, ***does an increase in the complexity of the embryo provasculature pattern lead to an increase in Fe content in the seed?*** In other words, we hypothesize that as consequence of increasing the proportion of cotyledons with four areoles or more, the volume of provasculature and endodermis will also be increased, and therefore iron content will be higher (**Figure 3**). Considering that overaccumulation of free Fe might lead to toxicity by production of reactive oxygen species (Ravet and Pilon, 2013), increasing iron content in seeds through the increase of the number of endodermal cells where iron accumulates in vacuoles can prevent oxidative stress. The mechanisms that control provasculature pattern complexity in Arabidopsis are not well defined, however, it has been described that *OPS* or *VCC* overexpressing plants produce seeds with increased vein pattern complexity in cotyledons (Truernit et al., 2012; Roschzttardtz et al., 2014). Overexpression of *OPS* leads to premature phloem differentiation, suggesting that phloem development could positively affect vascular patterning (Truernit et al., 2012). One factor involved in vascular patterning is auxin (Scarpella et al., 2010). However, in *VCC* overexpressing plants, changes in the provascular pattern complexity seem to be not related to auxin transport (Roschzttardtz et al., 2014).

In order to address our hypothesis, analysis of Fe content and distribution in different genotypes will be relevant. For instance, seeds from *VCC* and *OPS* overexpressing plants showed an increase of provasculature pattern complexity (Truernit et al., 2012; Roschzttardtz et al., 2014) and should be an excellent material to be used as proof of concept. In an opposite way, a decrease in provasculature complexity should lead to the production of seeds with less Fe content. If our hypothesis is correct, seeds from *vcc ops* double mutant plants that have a less complex provasculature pattern in cotyledons (Roschzttardtz et al., 2014), should have a significant decrease in total Fe content. An estimation of the possible iron increasing in cotyledon is shown in **Table 1**. In the case of a distribution of vein complexity like proposed for a hypothetical mutant shown in **Figure 3**, the total iron content could increase around to 26% in cotyledons.

**Table 1:** Estimation of iron increase in cotyledons with high provasculature complexity.

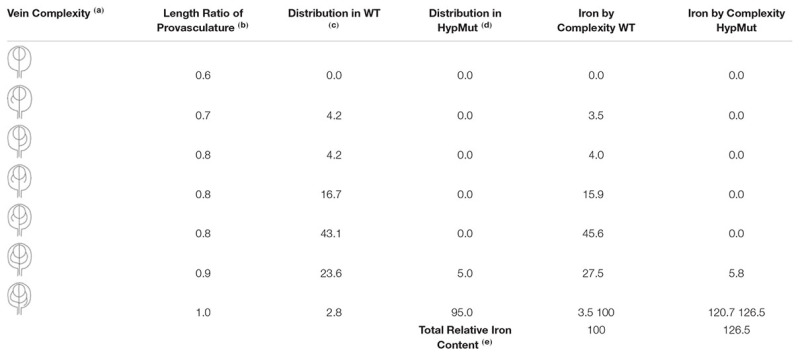

^(a)^*Vein provasculature complexity defined according to Roschzttardtz et al. (2014). Category “others” was not included*.

^(b)^*Length ratio of provasculature in cotyledons of 4–1’s category*.

^(c)^*Percentage of cotyledons according to Roschzttardtz et al. (2014)*.

^(d)^*Percentage of cotyledons according to **Figure 3***.

^(e)^*Only cotyledons*.

Elucidating the genetic control and signaling involved in the provasculature pattern complexity could be an important step in order to develop strategies to increased iron content in seeds.

## Concluding Remarks

In the Arabidopsis embryo, iron accumulates in the endodermis that surrounds the provasculature. We hypothesize that as an indirect consequence of increasing of provasculature pattern complexity, Fe content in seeds will also increase. This could be used as concept to develop strategies aimed to biofortify seeds.

## Author Contributions

All authors listed have made a substantial, direct and intellectual contribution to the work, and approved it for publication.

## Conflict of Interest Statement

The authors declare that the research was conducted in the absence of any commercial or financial relationships that could be construed as a potential conflict of interest.

## References

[B1] Carland F. Defries A. Cutler S. Nelson T. (2016). Novel vein patterns in *Arabidopsis* induced by small molecules. *Plant Physiol.* 170 338–353. 10.1104/pp.15.01540 26574596 PMC4704596

[B2] Carland F. Fujioka S. Takatsuto S. Yoshida S. Nelson T. (2002). The identification of CVP1 reveals a role for sterols in vascular patterning. *Plant Cell* 14 2045–2058. 10.1105/tpc.003939 12215504 PMC150754

[B3] Cnops G. Neyt P. Raes J. Petrarulo M. Nelissen H. Malenica N. (2006). The TORNADO1 and TORNADO2 genes function in several patterning processes during early leaf development in *Arabidopsis thaliana*. *Plant Cell* 18 852–866. 10.1105/tpc.105.040568 16531491 PMC1425859

[B4] DeFries R. Fanzo J. Remans R. Palm C. Wood S. Anderman T. L. (2015). Metrics for land-scarce agriculture. *Science* 349 238–240. 10.1126/science.aaa5766 26185232

[B5] Fan M. Zhao F. Fairweather-Tait S. Poulton P. Dunham S. McGrath S. (2008). Evidence of decreasing mineral density in wheat grain over the last 160 years. *J. Trace Elem. Med. Biol.* 22 315–324. 10.1016/j.jtemb.2008.07.002 19013359

[B6] Grillet L. Ouerdane L. Flis P. Hoang M. Isaure M. Lobinski R. (2014). Ascorbate efflux as a new strategy for iron reduction and transport in plants. *J. Biol. Chem.* 289 2515–2525. 10.1074/jbc.M113.514828 24347170 PMC3908387

[B7] Guerinot M. Yi Y. (1994). Iron: nutritious, noxious, and not readily available. *Plant Physiol.* 104 815–820. 10.1104/pp.104.3.815 12232127 PMC160677

[B8] Kim S. Punshon T. Lanzirotti A. Li L. Alonso J. Ecker J. (2006). Localization of iron in *Arabidopsis* seed requires the vacuolar membrane transporter VIT1. *Science* 314 1295–1298. 10.1126/science.1132563 17082420

[B9] Mary V. Schnell Ramos M. Gillet C. Socha A. Giraudat J. Agorio A. (2015). Bypassing iron storage in endodermal vacuoles rescues the iron mobilization defect in the natural resistance associated-macrophage protein3natural resistance associated macrophage protein4 double mutant. *Plant Physiol.* 169 748–759. 10.1104/pp.15.00380 26232490 PMC4577389

[B10] Murgia I. Arosio P. Tarantino D. Soave C. (2012). Biofortification for combating ‘hidden hunger’ for iron. *Trends Plant Sci.* 17 47–55. 10.1016/j.tplants.2011.10.003 22093370

[B11] Ravet K. Pilon M. (2013). Copper and iron homeostasis in plants: the challenges of oxidative stress. *Antioxid. Redox. Signal.* 19 919–932. 10.1089/ars.2012.5084 23199018 PMC3763233

[B12] Roschzttardtz H. Conejero G. Curie C. Mari S. (2009). Identification of the endodermal vacuole as the iron storage compartment in the *Arabidopsis* embryo. *Plant Physiol.* 151 1329–1338. 10.1104/pp.109.144444 19726572 PMC2773051

[B13] Roschzttardtz H. Conejero G. Curie C. Mari S. (2010). Straightforward histochemical staining of Fe by the adaptation of an old-school technique: identification of the endodermal vacuole as the site of Fe storage in *Arabidopsis* embryos. *Plant Signal. Behav.* 5 1–2. 10.4161/psb.5.1.10159 20592810 PMC2835959

[B14] Roschzttardtz H. Paez-Valencia J. Dittakavi T. Jali S. Reyes F. Baisa G. (2014). The vasculature complexity and connectivity (VCC) gene encodes a plant-specific protein required for embryo provasculature development. *Plant Physiol.* 166 889–902. 10.1104/pp.114.246314 25149602 PMC4213116

[B15] Scarpella E. Barkoulas M. Tsiantis M. (2010). Control of leaf and vein development by auxin. *Cold Spring Harb. Protoc.* 2:a001511 10.1101/cshperspect.a001511 PMC2827905 20182604

[B16] Sieburth L. Deyholos M. (2006). Vascular development: the long and winding road. *Curr. Opin. Plant Biol.* 9 48–54. 10.1016/j.pbi.2005.11.008 16332447

[B17] Sieburth L. Muday G. King E. Benton G. Kim S. Metcalf K. (2006). Scarface encodes an ARF-GAP that is required for normal auxin efflux and vein patterning in *Arabidopsis*. *Plant Cell* 18 1396–1411. 10.1105/tpc.105.039008 16698946 PMC1475492

[B18] Truernit E. Bauby H. Belcram K. Barthélémy J. Palauqui J. (2012). Octopus, a polarly localised membrane-associated protein, regulates phloem differentiation entry in *Arabidopsis thaliana*. *Development* 139 1306–1315. 10.1242/dev.072629 22395740

[B19] Vasconcelos M. Gruissem W. Bhullar N. (2016). Iron biofortification in the 21st century: setting realistic targets, overcoming obstacles, and new strategies for healthy nutrition. *Curr. Opin. Biotechnol.* 44 8–15. 10.1016/j.copbio.2016.10.001 27780080

[B20] Waters B. Grusak M. (2008). Quantitative trait locus mapping for seed mineral concentrations in two *Arabidopsis thaliana* recombinant inbred populations. *New Phytol.* 179 1033–1047. 10.1111/j.1469-8137.2008.02544.x 18631293

